# Soluble Urokinase Plasminogen Activator Receptor and the Risk of Coronary Artery Disease in Young Chinese Patients

**DOI:** 10.1155/2017/4719403

**Published:** 2017-10-04

**Authors:** Yuli Huang, Haobin Zhou, Yu Wu, You Yang, Wensheng Li, Jianhua Lu, Yunzhao Hu

**Affiliations:** ^1^Department of Cardiology, Shunde Hospital, Southern Medical University, Foshan, China; ^2^Department of Cardiology, Nanfang Hospital of Southern Medical University, Guangzhou 510515, China

## Abstract

**Background:**

Soluble urokinase plasminogen activator receptor (suPAR) is a novel marker of chronic inflammation and is considered to be a risk factor for coronary artery disease (CAD) in Caucasians. This study investigated the role of suPAR in young Chinese patients with CAD.

**Methods:**

The study involved a total of 196 consecutive young (age ≤ 55 years) patients with angiographically proven CAD and 188 age-matched non-CAD individuals as controls. Traditional risk factors were evaluated using conventional assays, and levels of suPAR were measured by sandwich enzyme-linked immunosorbent assays.

**Results:**

Levels of suPAR were significantly correlated with age (*r* = 0.20, *P* = 0.04), smoking (*r* = 0.33, *P* = 0.008), body mass index (*r* = 0.21, *P* = 0.03), and high-sensitivity C-reactive protein (hs-CRP; *r* = 0.31, *P* = 0.01). Multivariate logistic regression analysis showed that male sex (odds ratio (OR) = 3.12; 95% confidence interval (CI) = 1.18–8.25, *P* = 0.02), smoking (OR = 3.41, 95% CI = 1.55–7.50, *P* = 0.002), triglyceride (OR = 1.89, 95% CI = 1.10–3.25, *P* = 0.02), high-sensitivity C-reactive protein (OR = 1.24, 95% CI = 1.02–0.03, *P* = 0.03), and suPAR (OR = 1.37, 95% CI = 1.09–1.72, *P* = 0.007) were independently associated with CAD risk in young patients.

**Conclusions:**

SuPAR is a novel independent risk factor for CAD in young Chinese patients. Further studies evaluating the effect of anti-inflammatory treatment on the suPAR levels and the risk of CAD are needed.

## 1. Introduction

Coronary artery disease (CAD) is a major cause of mortality worldwide [1, 2]. Although CAD is generally associated with older age, it has recently been reported more frequently in younger individuals [3]. It has been reported that nearly 23% of patients with acute coronary syndrome (ACS) are under 55 years old [4]. The prevalence of CAD in younger patients has become common in China during the past decade, causing a great burden on society and health care costs [5].

The risk factors for CAD in young patients are different from those in older patients [3]. Traditional risk factors, such as hypertension, diabetes mellitus (DM), and hypercholesterolemia, are less prevalent in young patients with CAD. A recent study of myocardial infarction patients showed that 36% of young patients had none, or only one, traditional risk factor for CAD [6]. These patients would have been classified as low risk according to existing risk scoring systems of cardiovascular disease (CVD), such as the Framingham Risk Score (FRS). Therefore, there is a need for identifying other novel CAD risk factors in young patients.

The urokinase plasminogen activator receptor (uPAR) is a three-domain membrane-bound receptor [7]. It is expressed on several immune cells, endothelial cells, and smooth muscle cells and is involved in several immune functions, including adhesion, migration, angiogenesis, fibrinolysis, and cell proliferation [8]. Cleavage of the membrane-bound uPAR during inflammatory stimulation releases the soluble form, suPAR, into the circulation. The level of suPAR has been considered as a marker of immune activation and low-grade inflammation [9]. Recent studies have shown that suPAR is associated with an increased risk of CVD and mortality in the general population [10–13]. Furthermore, the Danish MONICA10 (monitoring trends and determinants of cardiovascular disease) study showed that the association between elevated suPAR and increased risk of CVD was stronger in younger individuals [14]. However, most of the evidence was from the Caucasian populations and there have only been few reports of the association of suPAR with the risk of CAD in Chinese populations. Therefore, this study aimed to evaluate the association between levels of suPAR and the risk of CAD in young Chinese patients, after adjusting for traditional CAD risk factors and FRS.

## 2. Materials and Methods

### 2.1. Subjects

The study complied with the Declaration of Helsinki and was approved by the Ethics Committee of Shunde Hospital, Southern Medical University, China. Written informed consent was obtained from all participants. In accordance with the previous reports, young patients with CAD were defined as those presenting with initial CAD symptoms at ≤55 years of age [15, 16]. A total of 196 young patients diagnosed with CAD by coronary angiography (CAG) were enrolled between September 2012 and September 2015. CAG was performed using the standard Judkins technique through the femoral or radial artery with an Allura Xper FD20 (Philips, Amsterdam, Netherlands). CAD was defined as ≥50% stenosis of the lumen diameter in at least one major coronary artery, including the left main coronary artery, left anterior descending branch, left circumflex branch, and right coronary artery. Two independent interventional cardiologists evaluated coronary artery stenosis.

A total of 188 age-matched individuals with normal CAG or negative findings by ultrafast coronary computed tomography angiography (CCTA) were enrolled as controls. Coronary angiography or CCTA was performed because of chest discomfort with suspected ischemic findings, such as ST deviation in an electrocardiograph, regional dyskinesia by echocardiography, or difficulty in differential diagnosis. Patients with uncontrolled infectious diseases, autoimmune diseases, hormone replacement therapy after menopause, malignancy, severe renal dysfunction (creatinine ≥ 3 mg/dL), and psychiatric disorders were excluded.

### 2.2. Laboratory Measurements

Venous blood samples were collected after overnight fasting. Levels of fasting plasma glucose, total cholesterol (TC), triglyceride (TG), high-density lipoprotein cholesterol (HDL-C), and serum creatinine (SCr) were measured using an Olympus AU2700 automatic biochemical analyzer (Japan). Low-density lipoprotein cholesterol (LDL-C) was calculated using the Friedewald equation. We used the high-sensitivity nephelometric method to determine the levels of hs-CRP. After centrifugation at 1500 ×g for 10 min at 4°C, plasma samples were stored at −80°C for future measurements of levels of suPAR. Levels of suPAR were measured using the commercial ELISA suPARnostic kit (ViroGates, Copenhagen, Denmark) according to the manufacturer's instructions. The interassay and intra-assay coefficients of variation were 6.2% and 4.3%, respectively. It has previously been shown that suPAR is highly stable throughout several freezing and thawing cycles [17].

### 2.3. Definition of Conventional Risk Factors for CAD

Conventional CAD risk factors evaluated in this study included the following: (1) Positive family history of premature CAD was defined as a diagnosis of CAD in a first-degree male relative < 55 years old or first-degree female relative < 65 years old. (2) With regard to smoking status, participants were classified as current smokers if they reported smoking regularly during the preceding year. Subjects who had never smoked or had stopped smoking at least 1 year before enrollment were classified as nonsmokers. (3) Hypertension was defined as per the Joint National Committee on Prevention, Detection, Evaluation, and Treatment of High Blood Pressure (JNC) VII guidelines [18]. (4) Diabetes mellitus was defined based on the 2003 American Diabetes Association criteria [19]. (5) Dyslipidemia was defined as per the 2007 Guidelines for Prevention and Treatment of Dyslipidemia in Adults in China, including TC ≥ 200 mg/dL, LDL-C ≥ 130 mg/dL, HDL-C < 40 mg/dL, and/or TG ≥ 150 mg/dL, or a history of receiving antidyslipidemia agents [20]. (6) BMI was indicative of being overweight (24–27.9 kg/m^2^) or obese (≥28 kg/m^2^) as per Chinese criteria [21]. (7) The estimated glomerular filtration rate (eGFR) was calculated using the modified Modification of Diet in Renal Disease equation adapted for Chinese subjects, as eGFR = 186 × Scr ^−1.154^ × age^−0.203^ × 0.742 (female) × 1.233 (Chinese) [22].

Based on the conventional risk factors for CAD, we also calculated the absolute 10 year CAD event risk scores based on the FRS system modified by the National Cholesterol Education Program Adult Treatment Panel III (NCEP ATP III) guidelines [23].

### 2.4. Statistical Analysis

All statistical analysis was performed using IBM SPSS Statistics for Windows, Version 22.0 (IBM Corp., Armonk, NY, USA). Continuous variables are presented as median (interquartile range) or mean (standard deviation) as appropriate. Categorical variables are expressed as percentages. Continuous variables were compared using the Mann–Whitney *U* or Student's *t*-test after testing for normality using the Kolmogorov–Smirnov test. Categorical variables were compared using the chi-square or Fisher's exact test, as appropriate. Pearson correlation for normal variables was used to evaluate the associations between study parameters. Skewed variables were logarithmically transformed (suPAR and hs-CRP) and analyzed as a continuous variable per one standard deviation of the log values.

Multiple logistic regression analysis was performed to evaluate the risk factors for CAD. Age, smoking history, hypertension, DM, family history of CAD, BMI, TG, TC, HDL-C, LDL-C, hs-CRP, eGFR, and suPAR levels were set as independent variables in the regression model. Odds ratios (ORs) and 95% confidence intervals (CIs) were calculated. *P* values < 0.05 were considered statistically significant. Furthermore, collinearity diagnostic statistics were performed to test the multicollinearity (strong correlations among independent variables). Variance inflation factor values > 2.5 or tolerance < 0.4 may indicate concern for multicollinearity in logistic regression models.

## 3. Results

### 3.1. Clinical Characteristics of Patients

We screened 214 young patients diagnosed with CAD by CAG and 203 age-matched controls. Based on the predefined exclusion criteria, 17 patients and 15 controls were excluded (eight patients with CAD, five controls with severe renal dysfunction, two patients with CAD with severe hepatic insufficiency, six controls with a history of anxiety or depression, eight patients with CAD, and four controls with uncontrolled infectious diseases). Therefore, 196 young patients with CAD (142 men, 54 women) and 188 age-matched controls (101 men, 87 women) were ultimately enrolled. Of the young patients diagnosed with CAD, 48 were classified as having unstable angina, 86 as having ST-segment elevation myocardial infarction, and 62 as having non-ST-segment elevation myocardial infarction.  Table 1 shows the demographics and clinical characteristics of all participants.

Compared with the control group, the CAD group had a higher proportion of smoking, overweight/obesity, and dyslipidemia (*P* < 0.05), but the differences in the proportions of a positive family history of CAD, hypertension, and DM were not significant (all *P* > 0.05). The levels of TG, hs-CRP, and suPAR were significantly higher in CAD patients than in controls (all *p* < 0.05), but there were no significant differences in other cardiovascular risk factors between the control group and the CAD group (all *p* > 0.05) (Table 1).

### 3.2. Correlation between suPAR and Other Risk Factors for CAD

Table 2 shows correlations between suPAR and other clinical variables. Plasma levels of suPAR were significantly positively correlated with age (*r* = 0.20, *P* = 0.04), smoking status (*r* = 0.33, *P* = 0.008), BMI (*r* = 0.21, *P* = 0.03), and hs-CRP levels (*r* = 0.31, *P* = 0.01), but not with levels of FPG, SBP, DBP, TG, TC, HDL-C, LDL-C, or eGFR (all *P* > 0.05).

### 3.3. Levels of suPAR and Risk of CAD in Young Patients

Multivariate logistic regression analysis revealed that male sex (OR = 3.12, 95% CI = 1.18–8.25, *P* = 0.02), smoking (OR = 3.41, 95% CI = 1.55–7.50, *P* = 0.002), TG (OR = 1.89, 95% CI = 1.10–3.25, *P* = 0.02), hs-CRP (OR = 1.24, 95% CI = 1.02–0.03, *P* = 0.03), and suPAR (OR = 1.37, 95% CI = 1.09–1.72, *P* = 0.007) were independently associated with CAD risk in young patients (Table 3). Multicollinearity analysis showed that the variance inflation factor value was >2.5, and the tolerance factor was <0.4, which indicates that there was no obvious multicollinearity in the logistic regression models.

## 4. Discussion

To our knowledge, this is the first report of suPAR being an independent risk factor for CAD in young Chinese patients. This correlation was observed even after adjustment for traditional cardiovascular risk factors, including hypertension, smoking, DM, dyslipidemia, and hs-CRP levels.

The immune system plays an important role in the pathogenesis of atherosclerosis. Chronic low-grade inflammation is significantly associated with the risk of CVD, while hs-CRP is the most common recommended risk marker of inflammation in cardiovascular risk stratification and has been widely evaluated in epidemiological studies [24]. Our results indicate that levels of the novel inflammatory marker, suPAR, are positively correlated with hs-CRP levels. However, the association between suPAR and the risk of CAD in young Chinese patients is independent of hs-CRP levels. This is in agreement with a previous report by Ghasemzedah et al. who showed that suPAR, hs-CRP, fibrin degradation product, and heat shock protein-70 are independent risk factors for CAD [25]. Lyngbaek et al. have shown that CRP is positively associated with anthropometric measures, such as BMI and waist circumference, whereas suPAR is linked to endothelial dysfunction and atherosclerosis [26]. Taken together, the above results indicate that these biomarkers may each be involved in the activation of different pathophysiological pathways leading to CAD.

Compared with hs-CRP, suPAR may be a more reliable factor for evaluating the risk of CAD. First, the role of hs-CRP in inflammation is controversial and it has been suggested that hs-CRP is merely a passive risk marker rather than a risk factor [13, 27]. For example, a Mendelian randomization study indicated that hs-CRP is a risk marker and not a causal factor for ischemic vascular disease [28]. In contrast, many *in vivo* and *in vitro* studies have shown that suPAR is involved in the development of atherosclerosis [29, 30]. Second, CRP is a highly inducible acute-phase protein, whereas suPAR does not show major variation. Levels of suPAR showed an increase of 15% following acute myocardial infarction, compared with a 365% increase in hs-CRP levels [31]. Furthermore, suPAR levels have been shown to have minimal circadian variation, which makes it a more suitable candidate as a clinical biomarker [14].

The exact pathophysiological mechanisms underlying the association between suPAR and CAD are still unclear, but we suggest several possibilities. First, suPAR and its ligand are involved in many pathogenic pathways, including pericellular proteolysis and matrix degradation, plasminogen activation and fibrinolysis, cell adhesion, migration, and proliferation [32]. Second, the suPAR molecule has intrinsic chemotactic properties and modulates the ability of monocytes to migrate in response to other chemokines [33]. Third, suPAR may also be involved in the rupture of atherosclerotic plaques, which causes acute attacks of CAD [12].

It has previously been reported that lifestyle modification, such as smoking cessation, and weight loss, would lower the levels of plasma suPAR [34, 35]. However, our study found that the association between increased levels of suPAR and the risk of CAD in young patients could not be completely explained by traditional cardiovascular risk factors, including smoking and obesity. Therefore, other measures to lower suPAR levels and further lower the risk of CAD should be explored. Anti-inflammatory treatment with canakinumab, a therapeutic monoclonal antibody targeting interleukin-1*β*, led to a significantly lower rate of recurrent CVD than placebo [36]. It has been reported that suPAR can be released from endothelial cells following stimulation by interleukin-1*β* [37]. It is, however, unclear if the observed canakinumab's effect on CVD is mediated by lowering the levels of suPAR.

The current study has some limitations. First, the case-control design of the study could not definitely evaluate a potential risk factor since reverse causality may exist. However, suPAR levels have been shown to be within minimal variation after acute myocardial infarction [31], and hence reverse causality is limited. Second, most of the controls were defined as non-CAD by CCTA, but not CAG, because of the excellent negative predictive value of CCTA in the diagnosis of CAD [38].

## 5. Conclusions

We report that suPAR, a novel inflammatory marker, is positively associated with the risk of CAD in young Chinese patients. Further studies to evaluate the effect of anti-inflammatory treatment on suPAR levels and the risk of CAD are needed.

## Figures and Tables

**Table 1:** Demographic and clinical characteristics of CAD patients and controls.

	CAD group (*n* = 196)	Control group (*n* = 188)	*P* value
Age (years)	48.3 ± 9.3	49.8 ± 10.4	0.14
Men (*n* (%))	142 (72.4%)	92 (48.9%)	<0.0001
CAD family history (*n* (%))	15 (7.7%)	11 (5.9%)	0.55
Current smokers (*n* (%))	76 (38.8%)	31 (16.5%)	<0.0001
Hypertension (*n* (%))	49 (25.0%)	35 (18.6%)	0.14
Systolic blood pressure (mmHg)	124.1 ± 12.5	123.0 ± 12.8	0.39
Diastolic blood pressure (mmHg)	73.2 ± 9.1	71.6 ± 8.8	0.08
Diabetes mellitus (*n* (%))	23 (11.7%)	17 (9.0%)	0.41
Fasting blood glucose (mg/dl)	90.3 ± 17.8	92.3 ± 16.7	0.26
Dyslipidemia (*n* (%))	63 (32.1%)	41 (21.8%)	0.03
TC (mg/dl)	189.0 ± 38.1	184.7 ± 39.5	0.28
LDL-C (mg/dl)	129.3 ± 27.9	125.1 ± 26.0	0.13
HDL-C (mg/dl)	41.5 ± 13.8	43.3 ± 13.5	0.14
TG (mg/dl)	157.6 ± 29.4	146.5 ± 28.2	0.0002
Overweight/obesity (*n* (%))	61 (31.1%)	40 (21.3%)	0.04
Body mass index (kg/m^2^)	24.8 ± 5.3	23.7 ± 4.6	0.03
eGFR (mL/min/1.73 m2)	111.2 ± 35.0	115.4 ± 39.8	0.27
hs-CRP (mg/dl)	5.36 (1.24–11.65)	2.28 (0.75–7.21)	<0.0001
suPAR (ng/ml)	5.8 (1.3–11.8)	2.8 (1.0–6.5)	<0.0001
10-year CAD event risk (%)	5.8 ± 4.7	5.0 ± 4.0	0.07

Data are presented as mean (±SD), median (interquartile range), or percentages. CAD: coronary artery disease; eGFR: estimated glomerular filtration rate; TC: total cholesterol; TG: triglyceride; HDL-C: high-density lipoprotein-cholesterol; LDL-C: low-density lipoprotein-cholesterol; hs-CRP: high-sensitivity C-reactive protein; suPAR: soluble urokinase plasminogen activator receptor.

**Table 2:** Correlation of suPAR and other cardiovascular risk factors.

Variables	*r* value	*P* value
Age	0.20	0.04
Sex	−0.11	0.21
Smoking	0.33	0.008
SBP	0.09	0.78
DBP	0.17	0.12
FPG	−0.10	0.56
TC	0.15	0.11
LDL-C	0.19	0.09
HDL-C	−0.14	0.08
TG	0.18	0.12
BMI	0.21	0.03
eGFR	0.15	0.26
hs-CRP	0.31	0.01

suPAR and hs-CRP were skewed variables and logarithmically transformed. suPAR: soluble urokinase plasminogen activator receptor; SBP: systolic blood pressure; DBP: diastolic blood pressure; FPG: fasting blood glucose; TC: total cholesterol; LDL-C: low-density lipoprotein-cholesterol; HDL-C: high-density lipoprotein-cholesterol; TG: triglyceride; BMI: body mass index; eGFR: estimated glomerular filtration rate; hs-CRP: high-sensitivity C-reactive protein.

**Table 3:** Risk factors for CAD in young patients in multivariate logistic regression analysis.

Risk factors	OR	95% CI	*P* value
Sex (male versus female)	3.12	1.18–8.25	0.02
Age (per 10 years)	1.32	0.85–2.05	0.22
Smoking (yes versus no)	3.41	1.55–7.50	0.002
CAD family history (yes versus no)	1.10	0.58–2.09	0.77
BMI (≥24 kg/m^2^ versus <24 kg/m^2^)	1.34	0.97–1.85	0.08
DM (yes versus no)	1.21	0.82–1.76	0.33
Hypertension (yes versus no)	1.25	0.37–4.22	0.72
TG (≥1.7 mmol/L versus <1.7 mmol/L)	1.89	1.10–3.25	0.02
TC (≥5.18 mmol/L versus <5.18 mmol/L)	1.04	0.75–1.44	0.81
LDL-C (≥3.37 mmol/L versus <3.37 mmol/L)	1.17	0.88–1.56	0.28
HDL-C (≥1.04 mmol/L versus <1.04 mmol/L)	0.97	0.12–7.84	0.98
eGFR (per 10 mL/min/1.73 m^2^)	1.58	0.42–5.94	0.50
Log hs-CRP (per SD)	1.24	1.02–1.51	0.03
Log suPAR (per SD)	1.37	1.09–1.72	0.007

BMI: body mass index; CAD: coronary artery disease; CHD: coronary heart disease; DM: diabetes mellitus; CI: confidence interval; eGFR: estimated glomerular filtration rate; HDL-C: high-density lipoprotein cholesterol; hs-CRP: high-sensitivity C-reactive protein; LDL-C: low-density lipoprotein cholesterol; OR: odds ratio; suPAR: soluble urokinase plasminogen activator receptor; TC: total cholesterol; TG: triglyceride.
